# Drug-drug interaction of paroxetine on olanzapine and initial dosage optimization in patients with major depressive disorder based on population pharmacokinetics

**DOI:** 10.3389/fpsyt.2025.1538996

**Published:** 2025-05-13

**Authors:** Cun Zhang, Liang Chen, Yin-Yin Duan, Su-Mei He, Ya-Li Tian, Ying Gao, Dong-Dong Wang

**Affiliations:** ^1^ Department of Pharmacy, Xuzhou Oriental Hospital Affiliated to Xuzhou Medical University, Xuzhou, Jiangsu, China; ^2^ Jiangsu Key Laboratory of New Drug Research and Clinical Pharmacy & School of Pharmacy, Xuzhou Medical University, Xuzhou, Jiangsu, China; ^3^ Department of Pharmacy, The Affiliated Huaian NO.1 People’s Hospital of Nanjing Medical University, Huaian, Jiangsu, China; ^4^ Department of Pharmacy, Suzhou Research Center of Medical School, Suzhou Hospital, Affiliated Hospital of Medical School, Nanjing University, Suzhou, Jiangsu, China; ^5^ Department of Infection Diseases, Suzhou Research Center of Medical School, Suzhou Hospital, Affiliated Hospital of Medical School, Nanjing University, Suzhou, Jiangsu, China; ^6^ Department of Cardiology, Xuzhou Municipal Hospital Affiliated to Xuzhou Medical University, Xuzhou, Jiangsu, China

**Keywords:** drug-drug interaction, paroxetine, olanzapine, population pharmacokinetics, initial dosage, major depressive disorder

## Abstract

**Objective:**

Olanzapine is already used to treat patients with major depressive disorder; however, whether complex drug–drug interaction (DDI) has an effect on the pharmacokinetics of people using olanzapine and its initial dosage remains unknown. The present study aims to explore the effect of DDI on olanzapine.

**Methods:**

In total, 72 patients with major depressive disorder were included for analysis. Potential physiological and biochemical indices and other drug combination information were collected to explore the effect of clinical olanzapine concentrations by building a nonlinear mixed effect (NONMEM) model and to further simulate the optimal olanzapine initial dosage by use of the Monte Carlo method in patients with major depressive disorder.

**Results:**

Weight and combined use of paroxetine significantly affected olanzapine clearance. With the same weight, the clearance rates of olanzapine were 0.711:1 in patients with major depressive disorder with or without paroxetine. For the initial dosages, without paroxetine, the olanzapine administration dosages, 0.5 and 0.4 mg/kg/day were recommended for patients with major depressive disorder in the groups weighing 40 to 56 kg and 56 to 100 kg, respectively. With paroxetine, olanzapine administration dosages of 0.3 and 0.2 mg/kg/day were recommended for patients with major depressive disorder in the groups weighing 40 to 85 kg and 85 to 100 kg, respectively.

**Conclusions:**

This has been the first case to establish olanzapine population pharmacokinetics in patients with major depressive disorder. In addition, the present study innovatively clarified that paroxetine affected olanzapine population pharmacokinetics and initial dosage in patients with major depressive disorder.

## 1 Introduction

Major depressive disorder is characterized by widespread and lasting depression and loss of interest manifested as low mood, pessimism, and depression accompanied by memory loss, fatigue, gastrointestinal discomfort, cognitive impairment, and other symptoms, resulting in a decline in physical and social function (1, 2). The most dangerous clinical symptom of major depressive disorder is suicide; the rate of suicide is 20 times that of people without major depressive disorder (3). Previous studies have shown that major depressive disorder has become the second most common disease after cardiovascular disease (4). It significantly reduces the quality of life and not only increases the mental burden of individuals but also the incidence and mortality of other diseases, such as cardiovascular disease and diabetes, leading to an increase in medical costs and further aggravating the economic burden of society (5, 6). The recent consensus statement on treatment-resistant depression by Maina et al. contextualizes the challenges of treatment-resistant depression and the need for alternative strategies (7).

At this stage, drug therapy is the first choice for the treatment of major depressive disorder, and the commonly used drugs for major depressive disorder mainly include selective serotonin and noradrenaline reuptake inhibitors, monoamine oxidase inhibitors, tricyclic antidepressants, and multimodal drugs. Olanzapine is an atypical antipsychotic medication widely used in the treatment of various psychiatric disorders. Its primary indications include schizophrenia, bipolar disorder, anxiety, and depression. The sedative effect of olanzapine is significantly stronger than that of aripiprazole; the sedative effect of two aripiprazole tablets was equivalent to one olanzapine tablet at a clinical equivalent dosage. Studies have shown that olanzapine can provide more benefits in the multi-drug combination of major depressive disorder (8–13). Olanzapine could be used as an alternative to lithium as an option for patients with major depressive disorder who do not respond to paroxetine treatment (12). In addition, usage of an olanzapine-fluoxetine combination in major depressive disorder has been reported (14, 15). However, olanzapine is greatly affected by drug–drug interactions (DDI) in clinical practice, and variation in dosage or drug concentration levels easily affects efficacy or results in adverse reactions (16–19). Several drugs exhibit interactions with olanzapine, such as fluvoxamine, fluoroquinolones, antiretroviral drugs, propafenone and flecainide, fluoxetine, and duloxetine (20–25). How to identify the factors affecting olanzapine and formulate an appropriate olanzapine administration regime for patients with major depressive disorder has become an urgent problems in clinical practice.

Population pharmacokinetics employs a nonlinear mixed-effects model to quantitatively characterize the absorption, distribution, metabolism, and excretion processes of drugs within populations, analyze inter-individual variability in pharmacokinetic parameters, and investigate the impact of covariates. The present study aims to collect potential physiological and biochemical indices and drug combination information to explore the effect on clinical olanzapine concentrations and to further simulate the optimal olanzapine initial dosage by using population pharmacokinetics and the Monte Carlo method, innovatively clarifying how DDI affects olanzapine population pharmacokinetics and initial dosage in patients with major depressive disorder.

## 2 Methods

### 2.1 Data collection

We collected data on patients with major depressive disorder who were hospitalized and treated with olanzapine at Xuzhou Oriental Hospital affiliated to Xuzhou Medical University, between December 2020 and August 2023, retrospectively. The inclusion criteria were as follows: (i) patients with major depressive disorder, (ii) olanzapine treatment, (iii) carrying out therapeutic drug monitoring (TDM) for olanzapine regularly, and (iv) a detailed treatment plan. Potential physiological and biochemical indices (which were obtained from the patient’s medical record system, and the detection of these indicators was carried out by the hospital laboratory according to the clinical diagnosis and treatment path, conventional), drug combination information, and olanzapine concentrations were collected. The above research was approved by the Research Ethics Committee of the Xuzhou Oriental Hospital affiliated to Xuzhou Medical University (No.20220725011), where the requirement for written informed consent could be waived since the data were collected without patient identifiers.

### 2.2 Modeling

In the modeling process of this study, apparent oral clearance (CL/F), volume of distribution (V/F), and absorption rate constants [Ka, fixed at 0.861/h (26)] were taken into consideration. In addition, the olanzapine population pharmacokinetic model in patients with major depressive disorder was built up using non-linear mixed effect modeling (NONMEM, version 7, ICON Development Solutions, Ellicott City, MD, USA) software.

Equation 1 shows inter-individual variability:


(1) $$A_{i}=TV\left(A\right)\times exp\;\left(\eta_{i}\right)$$

A_i_ is the individual parameter value. TV(A) is the typical individual parameter value. η_i_ is the symmetrical distribution, which was a random term with zero mean and variance omega^2 (ω^2^).

Equation 2 shows the random residual variability:


(2) $$B_{i}=\;C_{i}+\;C_{i*}\epsilon_{1}+\;\epsilon_{2}$$

B_i_ is the observed concentration. C_i_ is the individual predicted concentration. ϵ_n_ is the symmetrical distribution, which was a random term with zero mean and variance sigma^2 (σ^2^).

Equation 3 shows the relationship of pharmacokinetic parameters with weight:


(3) $$D_{i}=D_{std}\times {\left(E_{i}/E_{std}\right)}^{F}$$

D_i_ is the i-th individual parameter. E_i_ is the i-th individual weight. E_std_ is the standard weight of 70 kg. D_std_ is the typical individual parameter whose weight was E_std_. F is the allometric coefficient: 0.75 for the CL/F and 1 for the V/F (27).

Equations 4, 5 show the pharmacokinetic parameters between continuous covariates and categorical covariates, respectively:


(4) $$G_{i}=TV\left(G\right)\times {\left(Cov_{i}/Cov_{m}\right)}^{\theta }$$

(5) $$G_{i}=TV\left(G\right)\times (1+\theta \times Cov_{i})$$

G_i_ is the individual parameter value. TV(G) is the typical individual parameter value. θ is the parameter to be estimated. Cov_i_ is the covariate of the i-th individual. Cov_m_ is the population median for the covariate.

The covariate model was constructed in a stepwise way. Potential covariates included physiological and biochemical indices and drug combinations. The objective function value (OFV) variation was covariate inclusion criteria, among which OFV decrease>3.84 (*P*<0.05) was defined as the inclusion standard, and OFV increase>6.63 (*P*<0.01) was defined as the exclusion standard.

### 2.3 Model validation

Observations *vs.* population predictions, observations *vs.* individual predictions, absolute value of weighted residuals of individual (│iWRES│) *vs.* individual predictions, weighted residuals *vs.* time, density *vs.* weighted residuals, quantiles of weighted residuals *vs.* quantiles of normal, and visual predictive check (VPC) of the model and individual plot were used to evaluate the final model. Besides, the bootstrap method was used to compare with the final model parameters.

### 2.4 Simulation

Initial dosage optimization of olanzapine in patients with major depressive disorder was carried out using Monte Carlo simulation, where the olanzapine therapeutic window was 20 to 80 ng/ml (28). The present study found that weight and the combined use of paroxetine significantly affected olanzapine clearance. Therefore, according to whether paroxetine was used in combination or not, and as a once-daily or a twice-daily olanzapine (split evenly into two dosages a day) dose, we simulated four different cases; every case had 1000 virtual patients with major depressive disorder, 10 dosages (0.1, 0.2, 0.3, 0.4, 0.5, 0.6, 0.7, 0.8, 0.9, and 1.0 mg/kg/day) for seven weight groups (40, 50, 60, 70, 80, 90, and 100 kg). In the present study, the probability of achieving the target concentration was selected as the evaluation criterion.

## 3 Results

### 3.1 Patient information

Demographic data of patients with major depressive disorder are shown in **Table 1**: 72 patients with major depressive disorder (the concentration samples per patient were 1-3), 17 male and 55 female, whose ages ranged from 16.00 to 87.90 years old and weights were from 40.00 to 92.00 kg. Drug combination in patients with major depressive disorder are shown in **Table 2**, including atorvastatin calcium tablets, alprazolam tablets, amlodipine besylate tablets, benzoxol hydrochloride tablets, buspirone hydrochloride tablets, clonazepam tablets, dexzopiclone, duloxetine hydrochloride enteric-coated capsules, enteric-coated aspirin, escitalopram oxalate tablets, irbesartan hydrochlorothiazide tablets, levodopa and benserazide tablets, lorazepam tablets, metoprolol succinate tablets, mirtazapine tablets, omeprazole enteric-coated capsules, oxazepam, paroxetine hydrochloride tablets, propranolol hydrochloride tablets, sertraline hydrochloride tablets, trazodone hydrochloride tablets, valsartan capsules, venlafaxine hydrochloride tablets, and zopiclone tablets.

**Table 1:** Demographic data of patients with major depressive disorder (n = 72).

Characteristic	Mean ± SD	Median (range)
Gender (men/women)	17/55	/
Age (years)	48.14 ± 20.94	52.08 (16.00-87.90)
Weight (kg)	61.83 ± 11.82	60.00 (40.00-92.00)
Albumin (g/L)	39.51 ± 3.41	39.90 (30.80-47.40)
Globulin (g/L)	26.83 ± 2.97	26.80 (21.00-40.00)
Alanine transaminase (IU/L)	52.72 ± 109.29	27.00 (5.00-900.00)
Aspartate transaminase (IU/L)	37.38 ± 48.98	24.00 (12.00-368.00)
Creatinine (μmol/L)	53.88 ± 12.18	53.00 (4.73-96.00)
Urea (mmol/L)	4.48 ± 1.12	4.55 (1.03-9.11)
Total protein (g/L)	66.34 ± 4.61	65.60 (57.70-77.30)
Total cholesterol (mmol/L)	4.66 ± 1.25	4.61 (1.18-9.72)
Triglyceride (mmol/L)	2.19 ± 1.60	1.62 (0.47-6.80)
Direct bilirubin (μmol/L)	2.43 ± 1.55	2.20 (0.50-10.90)
Total bilirubin (μmol/L)	8.44 ± 3.65	7.80 (2.50-21.90)
Hematocrit (%)	38.16 ± 3.47	37.60 (31.40-47.60)
Hemoglobin (g/L)	126.81 ± 13.04	125.00 (97.00-168.00)
Mean corpuscular hemoglobin (pg)	30.56 ± 1.75	30.40 (25.20-34.60)
Mean corpuscular hemoglobin concentration (g/L)	332.10 ± 9.63	333.00 (307.00-362.00)

**Table 2:** Drug combination in patients with major depressive disorder (n = 72).

Drug	Category	N	Drug	Category	N
Atorvastatin Calcium Tablets	0	65	Lorazepam Tablets	0	61
	1	7		1	11
Alprazolam Tablets	0	61	Metoprolol Succinate Tablets	0	69
	1	11		1	3
Amlodipine Besylate Tablets	0	67	Mirtazapine Tablets	0	63
	1	5		1	9
Benzoxol Hydrochloride Tablets	0	69	Omeprazole Enteric-Coated Capsules	0	69
	1	3		1	3
Buspirone Hydrochloride Tablets	0	56	Oxazepam	0	68
	1	16		1	4
Clonazepam Tablets	0	60	Paroxetine Hydrochloride Tablets	0	54
	1	12		1	18
Dexzopiclone	0	70	Propranolol Hydrochloride Tablets	0	70
	1	2		1	2
Duloxetine Hydrochloride Enteric-Coated Capsules	0	59	Sertraline Hydrochloride Tablets	0	60
	1	13		1	12
Enteric-Coated Aspirin	0	67	Trazodone Hydrochloride Tablets	0	70
	1	5		1	2
Escitalopram Oxalate Tablets	0	62	Valsartan Capsules	0	70
	1	10		1	2
Irbesartan Hydrochlorothiazide Tablets	0	68	Venlafaxine Hydrochloride Tablets	0	70
	1	4		1	2
Levodopa and Benserazide Tablets	0	69	Zopiclone Tablets	0	58
	1	3		1	14

Category, 0: without drug, 1: with drug; N, number of patients.

### 3.2 Modeling

The final model of olanzapine in patients with major depressive disorder was shown in Equations 6, 7:


(6) $$CL/F=19.6\times {\left(weight/70\right)}^{0.75}\times (1-0.289\times PAR)$$

(7) V/F=197×(weight /70)

CL/F represents apparent oral clearance. V/F represents apparent volume of distribution. PAR represents paroxetine; when patients took paroxetine, PAR was 1, otherwise PAR was 0.

### 3.3 Evaluation

**Figure 1:**
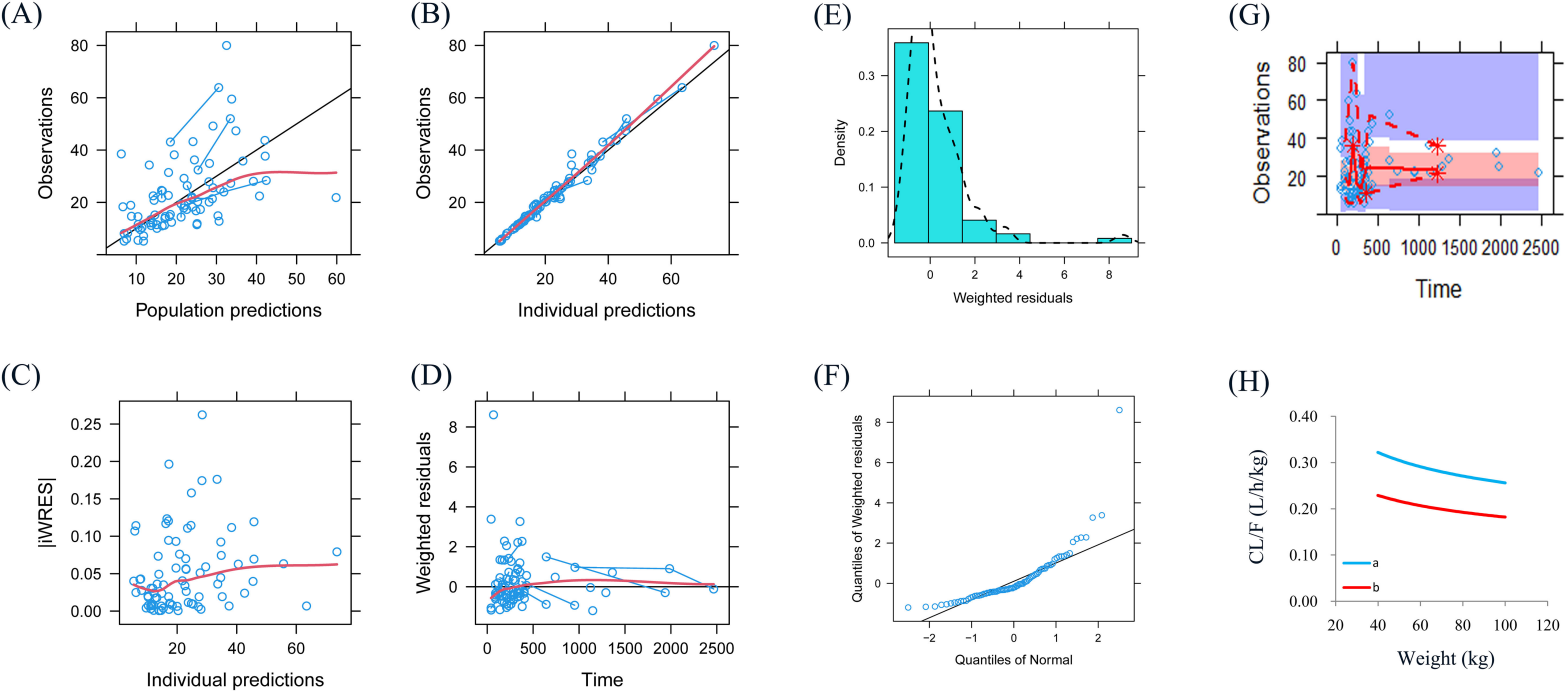
Model evaluation. **(A)** Observations *vs.* population predictions. **(B)** Observations *vs.* individual predictions. **(C)** absolute value of weighted residuals of individual (│iWRES│) *vs.* individual predictions. **(D)** Weighted residuals *vs.* time. **(E)** Density *vs.* weighted residuals. **(F)** Quantiles of weighted residuals *vs.* quantiles of normal. **(G)** Visual predictive check (VPC) of the model. **(H)** Olanzapine clearance. a: without paroxetine, b: with paroxetine.

**Figure 2:**
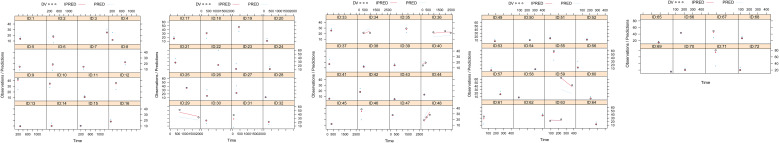
Individual plot. ID, patient ID number; DV, measured concentration value; IPRED, individual predictive value; PRED, population predictive value.

**Figures 1A-G** shows observations *vs.* population predictions, observations *vs.* individual predictions,│iWRES│*vs.* individual predictions, weighted residuals *vs.* time, density *vs.* weighted residuals, quantiles of weighted residuals *vs.* quantiles of normal, and VPC of the model. These results suggested that the final model predicted well. **Figure 1H** shows that with the same weight, the clearance rates of olanzapine were 0.711:1 in patients with major depressive disorder with or without paroxetine. **Figure 2** shows the individual plot, and from a clinical standpoint, our final model could predict the olanzapine concentrations of patients well at the individual level. **Table 3** shows the parameter estimate of the final model and bootstrap validation, indicating the final model was accurate and reliable.

**Table 3:** Parameter estimates and bootstrap validation in patients with major depressive disorder.

Parameter	Estimate	SE (%)	Bootstrap		Bias (%)
			Median	95% Confidence interval	
CL/F (L/h)	19.6	7.1	19.4	[17.0, 21.9]	-1.02
V/F (L)	197	15.3	194	[154, 277]	-1.52
Ka (h^-1^)	0.861 (fixed)	–	–	–	–
θ_PAR_	-0.289	30.5	-0.283	[-0.420, -0.078]	-2.08
ω_CL/F_	0.434	11.1	0.429	[0.336, 0.535]	-1.15
σ_1_	0.153	16.6	0.150	[0.061, 0.199]	-1.96
σ_2_	1.005	45.0	1.005	[0.306, 2.186]	0.00

95% confidence interval was displayed as the 2.5th, 97.5th percentiles of bootstrap estimates. CL/F, apparent oral clearance (L/h); V/F, apparent volume of distribution (L); Ka, absorption rate constant (h^-1^); θ_PAR_ was the coefficient of paroxetine; ω_CL/F_, inter-individual variability of CL/F; σ_1_, residual variability, proportional error; σ_2_, residual variability, additive error; Bias, prediction error, Bias = (Median-Estimate)/Estimate×100%.

### 3.4 Simulation

**Figure 3:**
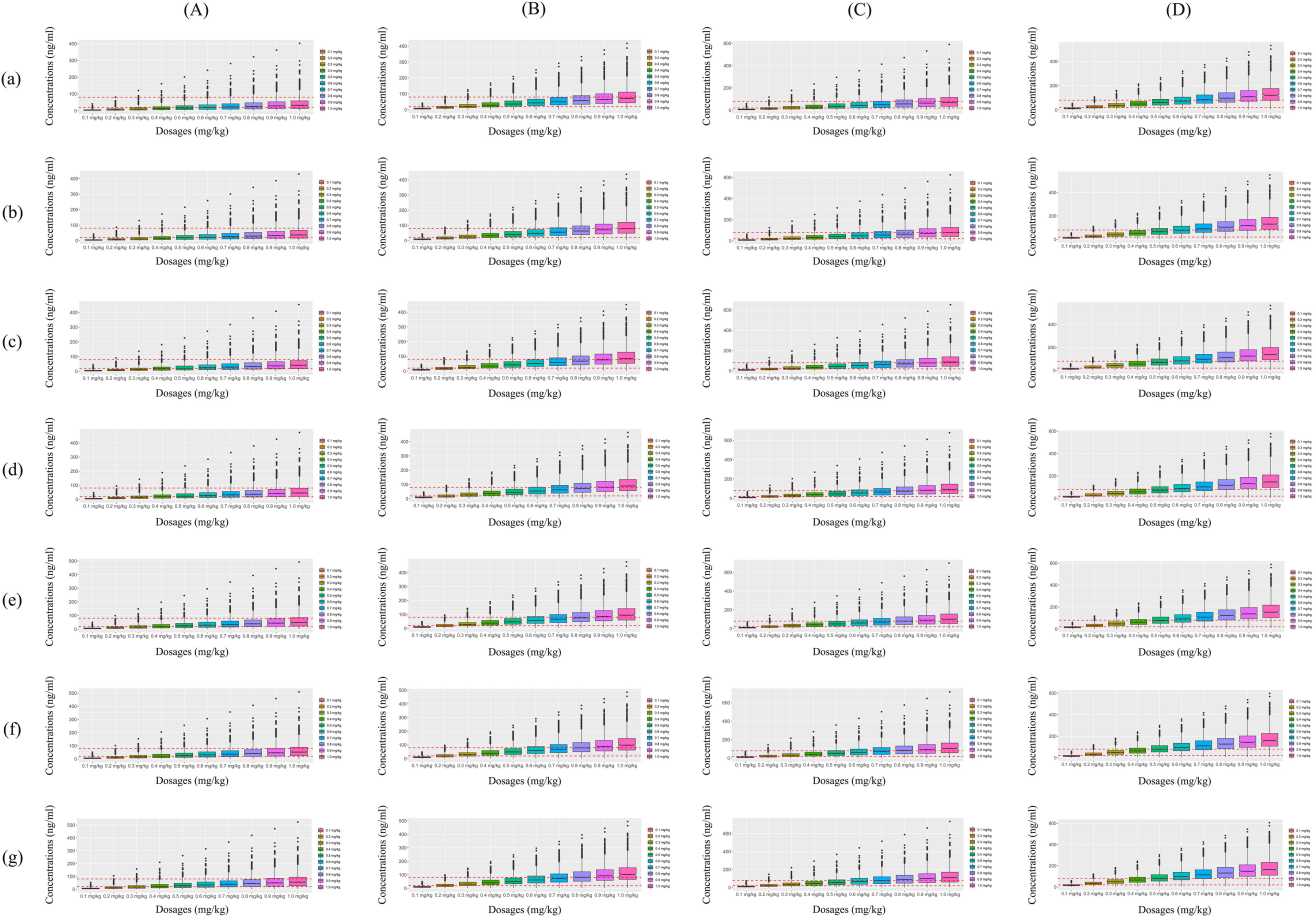
Simulated olanzapine concentrations. **(A)** Once-daily olanzapine administration dosages without paroxetine. **(B)** Twice-daily olanzapine administration dosages without paroxetine. **(C)** Once-daily olanzapine administration dosages with paroxetine. **(D)** Twice-daily olanzapine administration dosages with paroxetine. a: patients with major depressive disorder (40 kg), b: patients with major depressive disorder (50 kg), c: patients with major depressive disorder (60 kg), d: patients with major depressive disorder (70 kg), e: patients with major depressive disorder (80 kg), f: patients with major depressive disorder (90 kg), g: and patients with major depressive disorder (100 kg). The lower and upper red dashed lines were 20 and 80 ng/ml, respectively.

**Figure 4:**
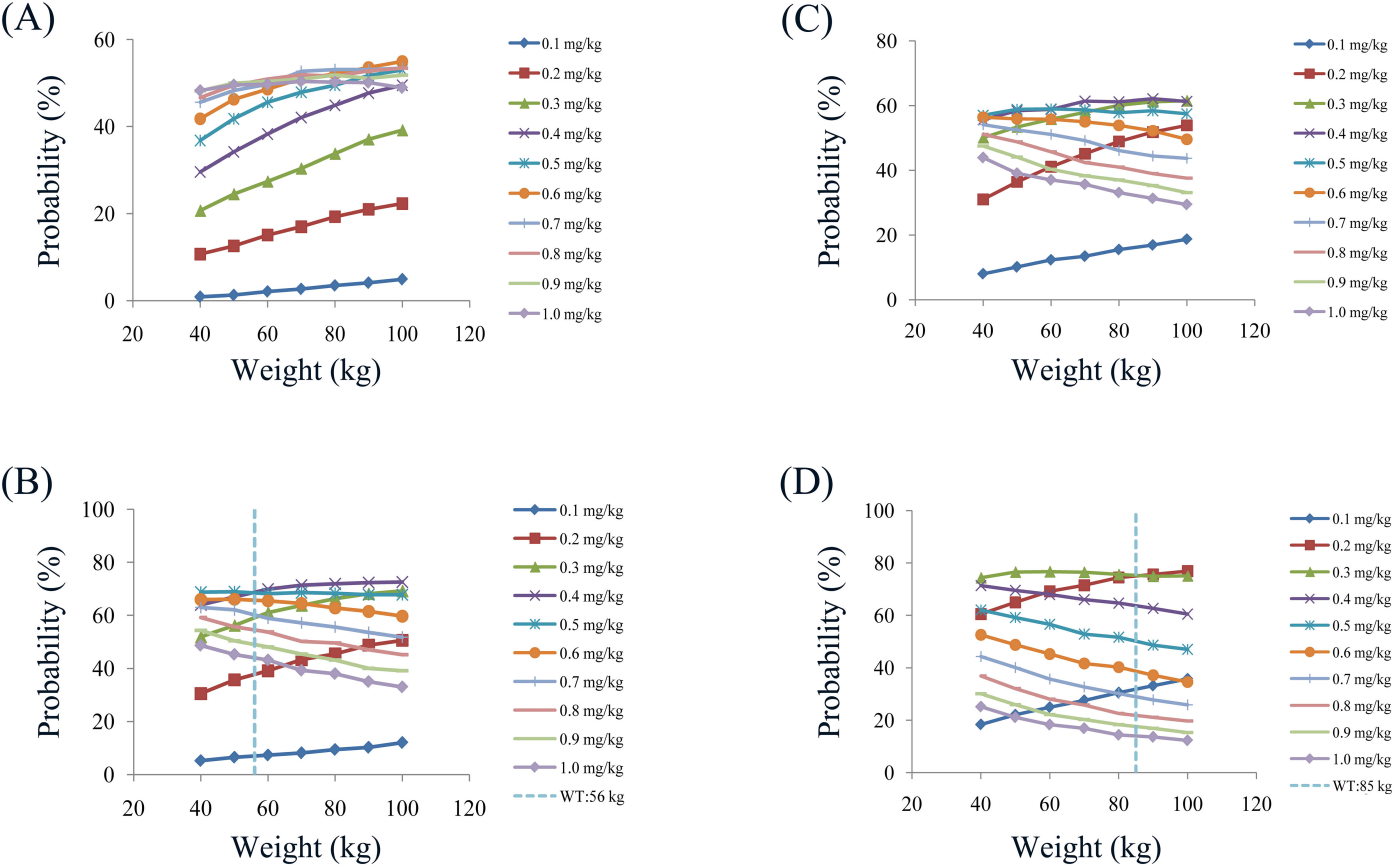
Probabilities for achieving a therapeutic window. **(A)** Once-daily olanzapine administration dosages without paroxetine. **(B)** Twice-daily olanzapine administration dosages without paroxetine. **(C)** Once-daily olanzapine administration dosages with paroxetine. **(D)** Twice-daily olanzapine administration dosages with paroxetine.

The simulated olanzapine concentrations of once-daily olanzapine administration dosages without paroxetine, twice-daily olanzapine administration dosages without paroxetine, once-daily olanzapine administration dosages with paroxetine, and twice-daily olanzapine administration dosages with paroxetine are shown in **Figures 3A–D**, respectively. Each colorful box diagram represents the predicted olanzapine trough levels of the corresponding dosage. The two red dashed lines represent the olanzapine therapeutic window (20–80 ng/ml), and the parts within the upper and lower red dashed lines represent concentrations reaching the therapeutic window. The probabilities of achieving the target concentration from once-daily olanzapine administration dosages without paroxetine, twice-daily olanzapine administration dosages without paroxetine, once-daily olanzapine administration dosages with paroxetine, and twice-daily olanzapine administration dosages with paroxetine are shown in **Figures 4A–D**, respectively. Based on simulation results, **Table 4** shows the optimal olanzapine initial dosages in patients with major depressive disorder. Without paroxetine, for once-daily olanzapine administration dosages, the probability for achieving the target concentrations from all dosages (0.1–1.0 mg/kg/day) was less than 55.0%. For twice-daily olanzapine administration dosages, 0.5 and 0.4 mg/kg/day were recommended for patients with major depressive disorder weighing 40 to 56 kg and 56 to 100 kg, respectively. Meanwhile, the probabilities of achieving target concentrations at these dosages were 68.5 to 68.9% and 68.5 to 72.6%, respectively. With paroxetine, for once-daily olanzapine administration dosages, 0.5 and 0.4 mg/kg/day were recommended for patients with major depressive disorder weighing 40 to 60 kg and 60 to 100 kg, respectively. Meanwhile, the probabilities of achieving target concentrations at these dosages were 57.0 to 59.0% and 58.8 to 62.1%, respectively. For twice-daily olanzapine administration dosages, 0.3 and 0.2 mg/kg/day were recommended for patients with major depressive disorder weighing 40 to 85 kg and 85 to 100 kg, respectively. Meanwhile, the probabilities of achieving target concentrations at these dosages were 74.4 to 76.7% and 75.1 to 76.9%, respectively.

**Table 4:** Initial dosage recommendation of olanzapine in patients with major depressive disorder with or without paroxetine.

Without paroxetine			With paroxetine		
Once a day			Once a day		
Body weight (kg)	Dose (mg/kg/day)	Probability of achieving the target concentrations (%)	Body weight (kg)	Dose (mg/kg/day)	Probability of achieving the target concentrations (%)
[40-100]	0.1-1.0	all ≤ 55.0	[40-60)	0.5	57.0-59.0
			[60-100]	0.4	58.8-62.1
Split evenly into two doses a day			Split evenly into two doses a day		
Body weight (kg)	Dose (mg/kg/day)	Probability of achieving the target concentrations (%)	Body weight (kg)	Dose (mg/kg/day)	Probability of achieving the target concentrations (%)
[40-56)	0.5	68.5-68.9	[40-85)	0.3	74.4-76.7
[56-100]	0.4	68.5-72.6	[85-100]	0.2	75.1-76.9

## 4 Discussion

The involvement of olanzapine in the treatment of patients with major depressive disorder has been widely reported (8–13), and researchers have shown that patients with major depressive disorder can receive more benefits from treatment with olanzapine. However, DDI may greatly affect the metabolism and formulation of the dosage regimen of olanzapine. In clinical practice, how to explore the influencing factors of olanzapine, quantify the degree of influence, and then formulate an optimal olanzapine dosage is urgent. TDM is guided by the basic theory of pharmacokinetics and pharmacodynamics, with the help of advanced analysis technology and electronic computer means, and the use of pharmacokinetic principles and formulas to individualize the drug delivery program (29–33). The blood concentration reported by TDM can provide a clinical basis for the next adjustment of the olanzapine administration schedule in patients. Nevertheless, due to the lack of blood concentration information, TDM alone cannot provide a reference for the initial dosage of olanzapine in patients with major depressive disorder.

Luckily, the combination of population pharmacokinetics and Monte Carlo simulation can make more full use of information from clinical TDM and provide references for initial drug administration recommendations through machine learning techniques. There has been considerable practice in this area, particularly focusing on DDIs. For example, Cai et al. found that voriconazole concomitant therapy affected tacrolimus in lung transplant recipients; meanwhile, the dosing regimen of tacrolimus was recommended based on whether voriconazole was combined (34). Chen et al. reported effects of posaconazole on tacrolimus population pharmacokinetics and initial dose in children with Crohn’s disease undergoing hematopoietic stem cell transplantation (35). Wang et al. reported effects of cimetidine on ciclosporin population pharmacokinetics and initial dose optimization in aplastic anemia patients (36). Chen et al. reported effects of voriconazole on population pharmacokinetics and optimization of the initial dose of tacrolimus in children with chronic granulomatous disease undergoing hematopoietic stem cell transplantation (37). Thus, the present study aims to explore the effect of DDI on olanzapine using population pharmacokinetics and Monte Carlo simulation.

In the present study, 72 patients with major depressive disorder were included, and potential physiological and biochemical indices and drug combination information were collected to explore the effect of olanzapine on clinical concentrations. Finally, weight and the combined use of paroxetine significantly affected olanzapine clearance. Paroxetine is a potent inhibitor of the CYP2D6 enzyme, and olanzapine is metabolized by the CYP2D6 enzyme, and then paroxetine inhibits the metabolism of olanzapine by inhibiting the CYP2D6 enzyme (38–44). With the same weight, the clearance rates of olanzapine were 0.711:1 in patients with major depressive disorder with or without paroxetine. Further, we simulated once-daily or twice-daily olanzapine administration dosages, among which twice daily was optimal. For the initial dosage of twice daily, without paroxetine, the olanzapine administration dosages 0.5 and 0.4 mg/kg/day were recommended for patients with major depressive disorder weighing 40 to 56 kg and 56 to 100 kg, respectively. With paroxetine, olanzapine administration dosages of 0.3 and 0.2 mg/kg/day were recommended for patients with major depressive disorder weighing 40 to 85 kg and 85 to 100 kg, respectively.

In addition, in a previous study, we used a similar research method to explore olanzapine population pharmacokinetics and initial dosage optimization in patients with schizophrenia, where 65 patients with schizophrenia were enrolled for analysis (45). In that study, we found that the combined use of aripiprazole significantly affected olanzapine clearance. Without aripiprazole, for twice-daily olanzapine administration dosages, 0.6 and 0.5 mg/kg/day were recommended for patients with schizophrenia weighing 40 to 60 kg and 60 to 100 kg, respectively. With aripiprazole, for twice-daily olanzapine administration dosages, 0.4 mg/kg/day was recommended for patients with schizophrenia weighing 40 to 100 kg (45). In summary, we have completed the precision administration and dosage recommendation of olanzapine in two independent populations: patients with schizophrenia and patients with major depressive disorder. In the future, we will further explore the precise administration and dosage recommendation of olanzapine in other populations.

Certainly, this study has limitations, such as the retrospective data, relatively small sample size, and insufficient in-depth exploration of patients’ dietary habits and comorbid diseases. Future research should conduct a prospective study with larger sample sizes and more comprehensive investigations into additional potential influencing factors.

## 5 Conclusion

This is the first study to establish olanzapine population pharmacokinetics in patients with major depressive disorder. In addition, the present study innovatively clarified that paroxetine affected olanzapine population pharmacokinetics and the initial dosage for patients with major depressive disorder.

## Data Availability

The original contributions presented in the study are included in the article/**Supplementary Material**. Further inquiries can be directed to the corresponding authors.

## Supplementary material

Supplementary Table 1 Effect of drug interaction and gender on olanzapine in patients with major depressive disorder.
