# Supplementary material for: Drug-drug interaction of paroxetine on olanzapine and initial dosage optimization in patients with major depressive disorder based on population pharmacokinetics
Source: Front Psychiatry. 2025 May 13;16:1538996. doi: 10.3389/fpsyt.2025.1538996 (PMC12107830; doi:10.3389/fpsyt.2025.1538996)
Supplement: Supplementary Table 1 — Effect of drug interaction and gender on olanzapine in patients with major depressive disorder. [file Table1.docx]

**Table S1. Effects of drug interaction and gender on olanzapine in major depressive disorder patients**

| **Forward inclusion 1** | | | | |
| --- | --- | --- | --- | --- |
| Model number | Instructions | OFV | Change of OFV | *P* value |
| 1 | Base model | 453.159 | 0 | / |
| 2 | Effect of Atorvastatin Calcium Tablets on CL | 452.38 | -0.779 | > 0.05 |
| 3 | Effect of Alprazolam Tablets on CL | 449.938 | -3.221 | > 0.05 |
| 4 | Effect of Amlodipine Besylate Tablets on CL | 447.224 | -5.935 | < 0.05 |
| 5 | Effect of Benzoxol Hydrochloride Tablets on CL | 453.048 | -0.111 | > 0.05 |
| 6 | Effect of Buspirone Hydrochloride Tablets on CL | 452.055 | -1.104 | > 0.05 |
| 7 | Effect of Clonazepam Tablets on CL | 450.847 | -2.312 | > 0.05 |
| 8 | Effect of Dexzopiclone on CL | 450.55 | -2.609 | > 0.05 |
| 9 | Effect of Duloxetine Hydrochloride Enteric-Coated Capsules on CL | 452.892 | -0.267 | > 0.05 |
| 10 | Effect of Enteric-Coated Aspirin on CL | 449.872 | -3.287 | > 0.05 |
| 11 | Effect of Escitalopram Oxalate Tablets on CL | 452.037 | -1.122 | > 0.05 |
| 12 | Effect of Irbesartan Hydrochlorothiazide Tablets on CL | 452.976 | -0.183 | > 0.05 |
| 13 | Effect of Levodopa and Benserazide Tablets on CL | 450.366 | -2.793 | > 0.05 |
| 14 | Effect of Lorazepam Tablets on CL | 453.045 | -0.114 | > 0.05 |
| 15 | Effect of Metoprolol Succinate Tablets on CL | 452.285 | -0.874 | > 0.05 |
| 16 | Effect of Mirtazapine Tablets on CL | 453.157 | -0.002 | > 0.05 |
| 17 | Effect of Omeprazole Enteric-Coated Capsules on CL | 451.675 | -1.484 | > 0.05 |
| 18 | Effect of Oxazepam on CL | 452.989 | -0.17 | > 0.05 |
| 19 | Effect of Paroxetine Hydrochloride Tablets on CL | 444.864 | -8.295 | < 0.05 |
| 20 | Effect of Propranolol Hydrochloride Tablets on CL | 452.009 | -1.15 | > 0.05 |
| 21 | Effect of Sertraline Hydrochloride Tablets on CL | 452.25 | -0.909 | > 0.05 |
| 22 | Effect of Trazodone Hydrochloride Tablets on CL | 452.801 | -0.358 | > 0.05 |
| 23 | Effect of Valsartan Capsules on CL | 453.143 | -0.016 | > 0.05 |
| 24 | Effect of Venlafaxine Hydrochloride Tablets on CL | 452.533 | -0.626 | > 0.05 |
| 25 | Effect of Zopiclone Tablets on CL | 451.389 | -1.77 | > 0.05 |
| 26 | Effect of Gender on CL | 450.03 | -3.129 | > 0.05 |
| **Forward inclusion 2** | | | | |
| Model number | Instructions | OFV | Change of OFV | *P* value |
| 27 | Model 19 | 444.864 | 0 | / |
| 28 | Model 19+Effect of Amlodipine Besylate Tablets on CL | 442.73 | -2.134 | > 0.05 |
| **Backward elimination** | | | | |
| Model number | Instructions | OFV | Change of OFV | *P* value |
| 29 | Model 19 | 444.864 | 0 | / |
| 30 | Model 19-Effect of Paroxetine Hydrochloride Tablets on CL | 453.159 | 8.295 | < 0.01 |

CL, clearance rate; OFV, objective function value.
